# Bioactive Molecules Released From Cells Infected with the Human Cytomegalovirus

**DOI:** 10.3389/fmicb.2016.00715

**Published:** 2016-05-13

**Authors:** Anna Luganini, Maria E. Terlizzi, Giorgio Gribaudo

**Affiliations:** Laboratory of Microbiology and Virology, Department of Life Sciences and Systems Biology, University of TurinTurin, Italy

**Keywords:** human cytomegalovirus, lytic infection, latency, bioactive molecules, secretome, chronic disease, angiogenesis, immunoevasion

## Abstract

Following primary infection in humans, the human cytomegalovirus (HCMV) persists in a latent state throughout the host’s lifetime despite a strong and efficient immune response. If the host experiences some form of immune dysregulation, such as immunosuppression or immunodeficiency, HCMV reactivates, thereby emerging from latency. Thus, in the absence of effective functional immune responses, as occurs in immunocompromised or immunoimmature individuals, both HCMV primary infections and reactivations from latency can cause significant morbidity and mortality. However, even in immunocompetent hosts, HCMV represents a relevant risk factor for the development of several chronic inflammatory diseases and certain forms of neoplasia. HCMV infection may shift between the lytic and latent state, regulated by a delicate and intricate balance between virus-mediated immunomodulation and host immune defenses. Indeed, HCMV is a master in manipulating innate and adaptive host defense pathways, and a large portion of its genome is devoted to encoding immunomodulatory proteins; such proteins may thus represent important virulence determinants. However, the pathogenesis of HCMV-related diseases is strengthened by the activities of bioactive molecules, of both viral and cellular origin, that are secreted from infected cells and collectively named as the secretome. Here, we review the state of knowledge on the composition and functions of HCMV-derived secretomes. In lytic infections of fibroblasts and different types of endothelial cells, the majority of HCMV-induced secreted proteins act in a paracrine fashion to stimulate the generation of an inflammatory microenvironment around infected cells; this may lead to vascular inflammation and angiogenesis that, in turn, foster HCMV replication and its dissemination through host tissues. Conversely, the HCMV secretome derived from latently infected hematopoietic progenitor cells induces an immunosuppressive extracellular environment that interferes with immune recognition and elimination of latently infected cells, thereby promoting viral persistence. Characterization of the composition and biological activities of HCMV secretomes from different types of infected cells will lay the foundation for future advances in our knowledge about the pathogenesis HCMV diseases and may provide targets for the development of novel antiviral intervention strategies.

## The Human Cytomegalovirus

Human cytomegalovirus (HCMV) is an opportunistic beta-Herpesvirus that infects more than 90% of people worldwide with an infection rate that increases with age (Landolfo et al., 2003; Britt, 2008; Mocarski et al., 2014). HCMV infection, as for the other human Herpesviruses, is characterized by two phases: lytic and latent. Following primary infection, in which the virus productively replicates in a broad range of different cell types (Sinzger et al., 2008), HCMV establishes latency in cells of myeloid lineage through a still poorly understood mechanism (Britt, 2008; Crough and Khanna, 2009; Mocarski et al., 2014). In the normal immunocompentent host, primary infection is usually asymptomatic or mildly symptomatic and persistent (Britt, 2008; Mocarski et al., 2014). Nevertheless, HCMV infection is considered, even in immunocompetent hosts, a risk factor for the development of various vascular diseases, immunosenescence, and tumor development (Britt, 2008; Mocarski et al., 2014; Nogalski et al., 2014). In contrast, in individuals in which the ability to develop an appropriate cellular immune response is compromised, such as immunosuppressed patients and the immunoimmature fetus during pregnancy, HCMV infection is one of the major causes of morbidity and mortality. Indeed, primary infection or reactivation from latency causes overt diseases in immunosuppressed hosts, such as transplant recipients taking immunosuppressive drugs and AIDS patients. In these settings, HCMV can be responsible for a wide range of clinical conditions, for example retinitis, pneumonia, colitis, hepatitis, and several chronic inflammatory diseases, such as atherosclerosis, transplant vascular sclerosis (TSV), and chronic allograft rejection (CR; Britt, 2008; Crough and Khanna, 2009; Mocarski et al., 2014). Congenital HCMV infections, on the other hand, represent the most prominent viral cause of birth defects such as malformations, hearing loss and learning disabilities (Kenneson and Cannon, 2007; Britt, 2008; Mocarski et al., 2014), and the incidence of HCMV transmission to the fetus is strictly related to maternal seroprevalence. Primary maternal infection carries a risk of transmission between 14.2 and 52.4% (Kenneson and Cannon, 2007), while a transmission rate of 1.4% has been reported in relation to maternal reactivated infections. However, the severity of congenital HCMV disease is similar for both primary and non-primary infections (Ahlfors et al., 2001; Rahav et al., 2007). Globally, the incidence of HCMV congenital infection is between 0.3 and 2.3% of all live births in developing countries. In congenitally infected fetuses, about 10–15% develops evident symptoms after birth, with an incidence of perinatal mortality of about 3–20 newborns per 100,000 live births, and of 24–160 cases of neurologic diseases (hearing loss and mental retardation) per 100,000 neonates. In the remaining 85–90% of HCMV-infected newborns, no symptoms are displayed at birth, but late signs of infection, such as hearing defects, are evident in 30–200 newborns per 100,000 live births. The impact of congenital HCMV infection on public health is thus significant, given that hearing loss, the most common long-term sequelae of infants with congenital HCMV, it is the leading cause of non-genetic deafness in children.

The broad range of clinical manifestations of HCMV diseases reflects the capacity of the virus to productively infect an extremely wide range of cell types in the host, such as skin and lung fibroblasts, epithelial and endothelial cells (ECs), vascular smooth muscle cells, hepatocytes, monocyte-derived macrophages, neuronal, and glial cells, thus determining its potential to spread to all areas of the body (Sinzger, 2008; Sinzger et al., 2008).

The HCMV genome is the largest among the Herpesviruses; its 235 kb double-stranded DNA is structured into a unique long (UL) and a unique short (US) region, both of which are flanked by terminal and internal inverted repeats (TRL/S and IRL/S, respectively; Landolfo et al., 2003; Mocarski et al., 2014). Even though its annotation remains provisional and its coding capacity has recently been proposed to be much greater than originally thought (Stern-Ginossar et al., 2012), it is generally accepted that the HCMV genome encodes at least 170 canonical proteins (Mocarski et al., 2014). Whole-genome functional profiling of two HCMV laboratory strains revealed that a set of just 50 herpesvirus-common proteins, encoded by genes mainly located in the central region of the U_L_ domain, is required for productive viral replication in primary fibroblasts (Murphy and Shenk, 2008; Mocarski et al., 2014). The remaining two-thirds of canonical HCMV protein-coding genes, mostly betaherpesvirus- or CMV-specific, are confined within the terminal regions of the genome and are not essential in cultured fibroblasts. Although, specific functions have yet to be assigned to many of these non-essential genes, they are mainly thought to be involved in regulating virus cell tropism, dissemination, and viral persistence and latency within the host, as well as the modulation of intrinsic, innate, and acquired host immune responses, thus contributing to viral pathogenesis in a variety of ways (Mocarski et al., 2014).

**FIGURE 1:**
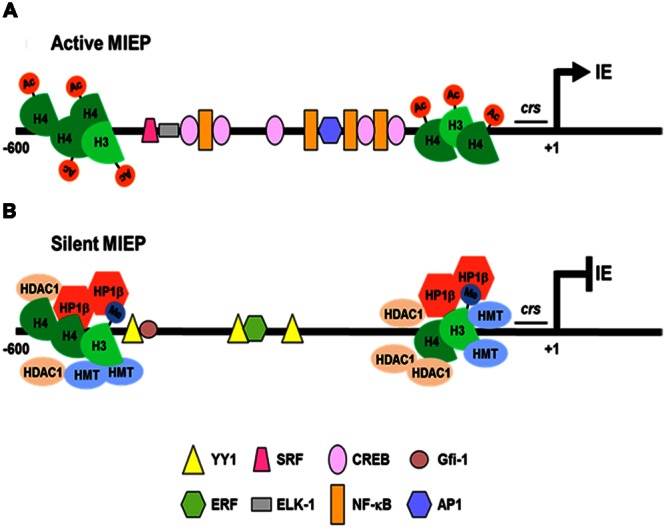
**Regulation of HCMV MIEP activity during lytic and latent infections. (A)** In cells permissive to lytic infection, the MIEP is associated with acetylated histones (Ac; H4 and H3) and many constitutive and inducible cellular transcription factors (NF-κB, CREB, AP1, SRF, and Elk-1) bind to cognate binding sites to activate IE genes transcription. **(B)** In latently infected cells, cellular transcriptional repressors (YY1, ERF, and Gfi-1) bind and recruit histone deacetylases (HDAC1) and methyltransferases (HMT) to the MIEP. The reduced content of acetylated H4 and the increase of dimethylated H3 histones (Me), promotes recruitment of the silencing protein (heterochromatin protein 1 -HP1β-), thus determining repression of the MIEP transcriptional activity.

During lytic infection, viral gene expression occurs in three phases, named immediate early (IE or α), early (E or β), and late (L or γ) in relation to their temporal kinetic profiles, and leads to genome replication, assembly and release of mature infectious viral particles. In brief, HCMV gene expression begins with *de novo* expression of IE genes, predominantly IE1-72 and IE2-86, that activate the expression of E genes, required for replication of the viral genome and the subsequent transcription of L (primarily structural) genes. IE2-86 protein autoregulates its own expression by negatively acting on the Major IE Promoter (MIEP) of HCMV (**Figure 1**; Stinski and Petrik, 2008); it binds to the *cis*-repressive sequence (CRS) of MIEP, resulting in a decrease in MIEP transcription (Stinski and Petrik, 2008). The activities of IE proteins, in turn, determine the expression of E genes, which are divided in two subgroups, β1 (E) and β2 (E-L), according to their time of expression; their functions are mainly related to HCMV replication machinery, viral DNA replication factors, repair enzymes, and immune evasion (Landolfo et al., 2003; Mocarski et al., 2014). L proteins are then expressed according to two different kinetics profiles (γ1 and γ2), shaped by distinct times of expression and their sensitivity to inhibitors of viral DNA replication. L proteins functions are associated to the assembly, maturation and egress of newly formed viral particles from the host cell.

In contrast to lytic replication, only a small subset of HCMV genes are expressed during latency (e.g., transcripts for the latency-associated viral IL-10/UL111A, LUNA [Latency Unique Natural Antigen], US28, UL138, and UL144) without any detectable production of infectious virus (Avdic et al., 2011; Reeves, 2011; Poole et al., 2014). HCMV latency is restricted to primary myeloid progenitors, such as granulocyte macrophage progenitors (GMPs), CD34^+^ hematopoietic progenitor cells, and CD14^+^ monocytes (Hahn et al., 1998; Scrivano et al., 2011; Rossetto et al., 2013). In these infected cell-types the carriage of viral DNA occurs in the absence of the expression of lytic genes (Mocarski et al., 2014). Only upon the terminal differentiation of these cells into macrophages or dendritic cells (DCs), HCMV lytic genes are expressed: IE gene products activate the expression of other genes, leading to viral DNA replication and *de novo* virus production (Taylor-Wiedeman et al., 1994; Hahn et al., 1998; Soderberg-Nauclér et al., 2001; Reeves et al., 2005; Britt, 2008; Huang et al., 2012; Mocarski et al., 2014). Since the number of cells carrying the latent viral genome is extremely low *in vivo* (about 0.001–0.01% of monocytes), it has been necessary to develop complex experimental latency models in order to study the related mechanisms. Thus, experimental HCMV latency and reactivation analyses have been performed using *in vitro* models that exploit primary myeloid progenitors cells: namely GMP cells, CD34^+^ hematopoietic cells, and CD14^+^ monocytes (Reeves et al., 2005; Cheung et al., 2006; Reeves and Sinclair, 2010; Liu et al., 2013). All of these studies have highlighted that a crucial aspect of HCMV latency is the repression of MIEP following its association with repressive chromatin markers (Bain et al., 2003; Wright et al., 2005; Sinclair and Sissons, 2006), involving the recruitment of histones and transcriptional silencing factors to the MIEP (Reeves, 2011). In addition to several binding sites for cellular transcription factors (e.g., NF-κB, CREB/ATF, AP1, SRF, Elk-1) that stimulate its transcriptional activity (**Figure 1**; Caposio et al., 2007a, 2010; Lashmit et al., 2009; Isern et al., 2011; Mocarski et al., 2014), the MIEP contains a series of multiple binding sites for transcription factors that may lead to its repression (YY1, ERF and Gfi-1; **Figure 1**; Reeves, 2011). In fact, during HCMV latency, YY1 and ERF bind to MIEP, and recruit histone deacetylases and methyltransferases than then target histones associated with MIEP (Wright et al., 2005). The methylated histones then become targets for the recruitment of heterochromatin protein 1 (HP-1), which augments MIEP repression and contributes to the establishment of latency (Liu et al., 2010). Therefore, permissive or latent infection may be determined by the balance between activating and repressive transcription factors that control MIEP activity (**Figure 1**; Reeves and Sinclair, 2013). However, this balance is thought to be mainly under the control of host inflammatory responses, either in immunocompetent or immunosuppressed individuals (Liu et al., 2013). Various studies have indeed shown that HCMV reactivation, during the allogeneic response to a transplanted organ, is mediated by the expression of inflammatory cytokines (i.e., TNF, LPS, and IL-6) that may, in turn, activate MIEP-interacting cellular transcription factors (e.g., NF-κB and AP-1), thus promoting MIEP activation and lytic HCMV gene expression (Hummel and Abecassis, 2002). However, in other experimental systems, the induction of IE gene expression has been associated with the state of cellular differentiation, as observed in non-permissive monocytes upon their differentiation into permissive macrophages in response to tumor necrosis factor (TNF) and IFN-γ (Soderberg-Nauclér et al., 1997).

## Modulation of Host Immune Responses By HCMV

Among the HCMV genes not essential for growth in cultured fibroblasts, about 40 have been shown to be involved in the modulation of host immune responses to viral infection by interfering with innate immune mechanisms and counteracting adaptive antibody and T-cell mediated immunity (Mocarski et al., 2014). The ability of HCMV to establish persistent infection, in spite of a robust T-cell and neutralizing antiviral antibody response, is mainly ensured by the activity of many virus-encoded immunomodulatory proteins. HCMV immunomodulation is therefore of pathogenetic importance in the establishment of virus persistence in the host. In this regard, HCMV is considered a master of immune evasion strategies, as highlighted by the results of several functional genomic, transcriptomic, and proteomic analyses that have defined many immunomodulatory functions of several HCMV proteins. In particular, these viral proteins have been observed to: (1) interfere with antigen presentation by major histocompatibility complex-I (MHC-I) molecules (e.g., the viral gene products US2, US3, US6, US10, US11, and UL82); (2) permit immune evasion through interference with and T and NK cells functions (e.g., the proteins encoded by genes UL16, UL18, UL40, UL83, UL141, UL142, US18, and US20); and (3) mimic the activities of cellular cytokines/chemokines (e.g., proteins UL111A, UL128, UL146, and UL147) or act as fake host cytokine/chemokine receptors (as observed for proteins UL144, UL21.5, US27, US28, UL33, and UL78; McSharry et al., 2012; Van Damme and Van Loock, 2014).

The best-characterized mechanisms of HCMV immunoevasion can be summarized as follows:

(1) Interference with antigen presentation by MHC-I molecules. A remarkable example is provided by glycoproteins US2, US3, US6, and US11, characterized by a single transmembrane (TM)-spanning and an immunoglobulin (Ig) domain, which most likely arose by duplication of a single viral ancestor gene (Gewurz et al., 2001). These HCMV glycoproteins, when expressed in fibroblasts, endothelial/epithelial cells, or DCs, induce, through different mechanisms, a decrease in the cell-surface expression of MHC-proteins required for the activation of CD8^+^ T lymphocytes (Mocarski, 2002). In particular, both US2 and US11 proteins are able to redirect nascent MHC class I proteins from the ER into the cytosol until ubiquitin-dependent proteasome-mediated degradation occurs. The US2 glycoprotein has also been observed to inhibit MHC class II protein translocation, thus inducing a substantial block of CD4^+^ T cell expansion. On the other hand, the US3 gene product prevents the egress of MHC class I proteins from the endoplasmic reticulum (ER) to the Golgi apparatus, whereas the US6 protein is able to bind the transporter of antigen processing (TAP) in the lumen of the ER, preventing ATP-dependent peptide loading on the cell membrane (Mocarski, 2002). In conclusion, this type of immunoevasion prevents the immune recognition of HCMV-infected cells.
(2) Immune evasion through interference with T and NK cells functions. Lytically HCMV-infected cells exhibit a noteworthy resistance to NK cell-mediated cytolysis *in vitro* (Wilkinson et al., 2008). HCMV is, in fact, able to alter and modify NK cell activity through different ‘evasion’ strategies, such as those exerted by: (i) UL16, a viral glycoprotein able to directly bind and sequester in the ER MICB, ULBP1, and ULBP2 -ligands for the activating NKG2D receptor present on NK cells- thus preventing their expression on the surface of HCMV-infected cells (Wilkinson et al., 2008); (ii) UL18, a viral-MHC-I homolog, which was found to bind the NK cell inhibitory receptor LIR1/ILT2 with a 1000-fold higher affinity than HLA-I molecules (Wilkinson et al., 2008); (iii) UL40, a viral protein able to promote the HLA-E upregulation on the surface of HCMV-infected cells. Since HLA-E acts as a NK cell-mediated cytotoxicity suppressor through the NK cell inhibitory receptor complex CD94/NKG2A (Prod’homme et al., 2012), its upregulation contributes to the NK cell immunoevasion of HCMV-infected cells; (iv) UL83, that encodes for the major HCMV tegument protein pp65 that suppresses the induction of several interferons (IFNs) and proinflammatory chemokine transcripts; pp65 may also directly bind to the activator receptor NKp30, present on the surface of NK cells, thus suppressing NK cell activation (Wilkinson et al., 2008); (v) UL141, a viral glycoprotein able to sequester CD155 molecule in the ER. CD155 expression leads to NK cell activation by binding to NK receptors CD226 and CD96 (Nemčovičová et al., 2013). Therefore, sequestration of CD155 prevents NK cell activation and survival of infected cells; (vi) US18 and US20 proteins recently observed to promote lysosomal MICA degradation (Fielding et al., 2014). MICA, the MHC class I polypeptide-related sequence A, is a natural ligand of the NKG2D receptor. NKG2D-MICA binding induces NK cell activation, leading to a cytolytic response against HCMV-infected cells. Therefore, MICA degradation reduces the capability of NK cells to recognize infected cells (Fielding et al., 2014).
(3) Mimic physiological activities of cellular cytokines/chemokines or host cytokine/chemokine receptors. HCMV encodes several homologs of chemokine/cytokine and/or their receptors that may interfere with the corresponding host counterparts. In this regard, an important example of a HCMV-encoded chemokine receptor homolog is represented by the multifunctional US28 protein. US28 is considered a putative immunoevasion molecule due to its capability to bind and internalize a range of cellular chemokines, including a broad spectrum of CC and CX3C chemokines, thus limiting the ability of host chemotactic molecules to elicit their effects in immune cells stimulation (Mocarski et al., 2014). However, US28 makes use of both the extracellular chemokine milieu and different intracellular G-protein pathways to produce a wide variety of pathophysiological cell responses (Beisser et al., 2008). In fact, upon stimulation with some chemokines, US28 specifically promotes smooth muscle cells and macrophage migration, thus contributing to both virus dissemination and pathogenesis of HCMV-associated vascular diseases (Vomaske et al., 2009). On the other hand, constitutive expression of US28 induces, in a ligand-independent manner, cyclooxygenase-2 (COX-2) expression via NF-κB activation, leading to the production of VEGF, one of the most abundant angiogenic factors (Maussang et al., 2009).

Moreover, the release of virus-encoded cytokines/chemokines homologs (see HCMV-Induced Secreted Cellular Proteins) permits the virus to modulate specific host immune defense mechanisms in infected tissues, since these bioactive soluble molecules act in a paracrine fashion.

## Bioactive Molecules Released From HCMV-Infected Cells

The pathogenesis of acute HCMV diseases is related to end-organ damage that results from both lytic virus replication and host immune responses, whereas diseases associated to persistent infections in both immunocompetent and immunocompromised patients (transplant recipients and AIDS patients) are related to chronic inflammation (Britt, 2008). However, besides the direct cytopathic effect of virus replication on host tissues, the pathogenesis of HCMV diseases may be influenced by the activity of virus-induced molecules secreted from virus-infected cells. These secreted factors, of both viral and cellular origin, by acting in a paracrine fashion alter and modify the local microenvironment, thus contributing to the development of HCMV-related diseases (Streblow et al., 2008).

### HCMV-Induced Secreted Cellular Proteins

Early *in vitro* studies on the ability of HCMV to stimulate the secretion of bioactive cellular factors identified several cytokines and growth factors released from different types of infected cells. First, Almeida et al. (1994) observed that HCMV infection of HUVECs (Human Umbilical Vein Endothelial Cells) greatly increased the expression of IL-6 mRNA and the secretion of this cytokine into the supernatant of infected cells. Dengler et al. (2000) went on to demonstrate the paracrine activity of IL-1β released from HCMV-infected cells, which resulted in an upregulation of pro-inflammatory adhesion molecules on non-infected neighboring cells. In fact, by analyzing the expression of plasma membrane proteins on the surface of both HCMV-infected HUVECs and human vascular smooth muscle cells (hvSMC), they observed a 200-fold overexpression of the Inter Cellular Adhesion Molecule-1 (ICAM-1) and *de novo* induction of both Vascular Cell Adhesion Molecule 1 (VCAM-1) and E-selectin (Dengler et al., 2000). In regard to the EC model, we observed an upregulation of ICAM-1, IL-8, CCL5/RANTES, CXCL10/IP-10, CXCL11/I-TAC, and COX-2 gene expression in HUVECs cells infected with a low-passage HCMV strain, thus indicating a direct involvement of HCMV in the modification of the extracellular vascular environment through the release of inflammatory mediators (Caposio et al., 2007b).

Using astrocyte and microglia cell models, Maxim et al. (2001) observed that HCMV stimulated the release of CCL2/MCP-1 and IL-8 in the supernatants of infected astrocytes, whereas infection of microglia cells led to an increased secretion of TNF-α, IL-6, CCL2/MCP-1, IL-8, CCL5/RANTES, and CCL3/MIP-1α. These findings thus suggest the ability of infected astrocytes to recruit microglia cells, through the release of the chemoattractant CCL2/MCP-1 (Maxim et al., 2001).

However, a different picture emerged from the investigation of the HCMV-mediated modulation of chemokine gene expression in the context of HCMV retinitis – a virus-induced inflammation of the retina characterized by vasculitis and retina degeneration that leads to retinal detachment in immunocompromised hosts. In the *in vitro* cell model provided by human retinal pigment epithelial cells, Momma et al. (2003) observed, by means of RT-PCR and ELISA assays, that HCMV infection caused an upregulation of IL-8, whereas CCL2/MCP-1 and CCL7/MCP-3 levels were downregulated. The authors thus suggested that the altered secretion of IL-8, CCL2/MCP-1 and CCL7/MCP-3 by retinal cells may be involved in the initiation and development of the inflammatory process responsible for the pathogenesis of HCMV retinitis. In this scenario, the increased secretion of IL-8 may stimulate the recruitment and trafficking of leukocytes to the site of infection within the retina; on the other hand, the decrease in CCL2/MCP-1 and CCL7/MCP-3 levels in the extracellular environment may limit the migration of leukocytes, thus contributing to the virus escape of the host innate immune response (Momma et al., 2003).

Together, these earlier studies demonstrated how HCMV is able to alter in a cell-type specific manner the extracellular microenvironment surrounding infected cells by stimulating the secretion of cellular bioactive factors.

However, only after the introduction of high-throughput large-scale proteomic approaches including antibody-based arrays and liquid chromatography-mass spectrometry (MS/LC), was it possible to drawn up an in-depth depiction of the complexity of the bioactive molecules released upon HCMV infection (Dumortier et al., 2008; Botto et al., 2011; Caposio et al., 2011, 2013; Fiorentini et al., 2011; Mason et al., 2012; MacManiman et al., 2014; Noriega et al., 2014; Gustafsson et al., 2015). These global biochemical characterizations confirmed the cell-type specific patterns of secreted cellular factors from HCMV-infected cells and most of the alterations of types and quantities of bioactive molecules already observed in earlier studies.

Thanks to proteomics studies, the entire array of bioactive proteins of present in supernatants from different types of HCMV-infected cells, has now been defined, qualitatively and quantitatively, and designated the HCMV secretome (Streblow et al., 2008).

**FIGURE 2:**
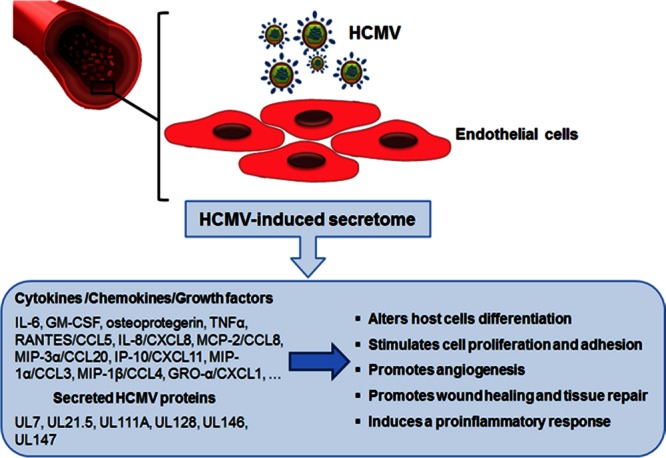
**The role of endothelial cell-derived HCMV secretome in accelerating vascular diseases.** Productive HCMV infection of endothelial cells leads to the release of many bioactive proteins that can modify the microenvironment around host cells. The ability of these secreted factors to promote neo-vessel formation, wound healing, and the inflammatory response may help explain the role of HCMV infection in the development of HCMV-associated vascular diseases.

In the first of these studies, Dumortier et al. (2008) analyzed virus-free supernatants from lytically infected fibroblasts (NHDF) to investigate the complexity and heterogeneity of the HCMV-induced secretome. Using gel-free liquid chromatography (LCQ)-MS-MS, they identified more than 1,200 proteins in the secretome of HCMV-infected cells. Among them, several factors involved in angiogenesis (AG) and wound healing (WH) were further confirmed using a wide range human cytokine antibody array (RayBio G Series 2000 arrays). A conspicuous number of cytokines (IL-5, IL-6, IL-1α, IL-1β, GM-CSF, osteoprotegerin, TNF-α, TNF-RI and -RII), growth factors (angiopoietin, angiotensinogen, FGF, GDNF, HGF, IGF-BP, osteopontin, PDGF, PIGF, SPARC, and VEGF), extracellular matrix proteins, chemokines (IL-8/CXCL8 and CXCL1/GRO-α), enzymes, and adhesion molecules were identified (**Table 1**). The biological effects of HCMV secretome-derived proteins on AG and WH were then assessed using a modified matrigel *in vitro* tubule formation assay and an electric WH assay, respectively (Dumortier et al., 2008). However, many of the identified factors in the NHDF-derived secretome are also involved in the inflammatory response and EC activation, thus leading to cell proliferation, adhesion, and inflammatory response, all of which are indeed linked to TVS pathogenesis (**Figure 2**; Dumortier et al., 2008; Streblow et al., 2008). Thus, it was suggested that HCMV infection alters the local microenvironment through the secretion of cellular factors from infected cells that, in turn, by acting in a paracrine fashion, may stimulate AG and WH, both associated to the development of vascular diseases, such as TVS (Streblow et al., 2008).

**Table 1:** The most abundant bioactive factors determined by Ray Biotech protein assay analysis in the secretome of lytic HCMV-infected cells.

				Cell types		
Classification	Symbol	Gene annotation	Biological function	NHDF^1^	HUVEC^2^	LEC^3^
Cytochine/chemochine	CXCL1; GROα	Chemokine (C-X-C motif) ligand 1	Chemoattractant for neutrophils	•	•	•
	CXCL5; ENA-78	Chemokine (C-X-C motif) ligand 5	Recruitment and activation of leukocytes	•		•
	CXCL6; GCP-2	Chemokine (C-X-C motif) ligand 6	Chemoattractant for neutrophilic granulocytes		•	
	CXCL9; MIG	Chemokine (C-X-C motif) ligand 9	Leukocyte trafficking			•
	CXCL10; IP-10	Chemokine (C-X-C motif) ligand 10	Stimulation of monocytes, natural killer, and T-cell migration	•	•	
	CXCL11; I-TAC	Chemokine (C-X-C motif) ligand 11	Chemoattractant	•	•	•
	CXCL16	Chemokine (C-X-C motif) ligand 16	Recruitment of leukocytes	•	•	
	CCL1; I-309	Chemokine (C-C motif) ligand 1	Leukocyte trafficking			•
	CCL3; MlP-1α	Chemokine (C-C motif) ligand 3	Inflammatory responses	•	•	
	CCL4; MlP-1β	Chemokine (C-C motif) ligand 4	Chemokinetic and inflammatory functions	•	•	•
	CCL5; RANTES	Chemokine (C-C motif) ligand 5	Chemoattractant for blood monocytes, memory T helper cells and eosinophils	•	•	
	CCL7; MCP-3	Chemokine (C-C motif) ligand 7	Chemoattractant for macrophages	•	•	•
	CCL8; MCP-2	Chemokine (C-C motif) ligand 8	Leukocyte trafficking		•	•
	CCL15; MIP-1δ	Chemokine (C-C motif) ligand 15	T-cells and monocyte chemoattractant			•
	CCL20; MIP-3α	Chemokine (C-C motif) ligand 20	Leukocyte trafficking	•	•	•
	CCL23; MPIF-1	Chemokine (C-C motif) ligand 23	Chemotactic activity on resting T lymphocytes and monocytes		•	
	IL-1α	Interleukin-1α	Role in immune responses and inflammatory processes			•
	IL-3	Interleukin-3	Role in cell growth, differentiation, and apoptosis			•
	IL-5	Interleukin-5	Growth and differentiation factor for B cells and eosinophils	•		•
	IL-6	Interleukin-6	Anti-inflammatory cytokine	•	•	•
	IL-8	Interleukin-8	Leukocyte trafficking	•	•	
	IL-10	Interleukin-10	Anti-inflammatory cytokine			•
	IL-13	Interleukin-13	Anti-inflammatory cytokine			•
	IL-15	Interleukin-15	T and natural killer cells activation and proliferation			•
	LTA; TNF-β	Lymphotoxin A	Proinflammatory cytokine			•
	TNF-α	Tumor necrosis factor alpha	Proinflammatory cytokine		•	•
	Osteoprotegerin	PG/TNFRSF11B	Lymph-node organogenesis and vascular calcification	•	•	
Growth factor	GM-CSF	Granulocyte-Macrophage Colony-Stimulating-Factor	Bone and cartilagine development	•	•	•
	bFGF	Fibroblast Growth Factor 2 (basic)	Mitogenic and angiogenic activities			•
	TGF-β1	Transforming growth factor beta 1	Cell growth, proliferation, differentiation, adhesion, and migration	•		•
	HGF	Hepatocyte growth factor	Angiogenesis	•		
Receptor	ICAM-1	Intercellular adhesion molecule 1	Cell adhesion for endothelial and immune system cells	•	•	•
	TNF-RI	Tumor necrosis factor receptor 1	Regulator of inflammation	•		•
	TNF-RII	Tumor necrosis factor receptor 2	Regulator of inflammation	•		
ECM	MMP-1	Matrix metallopeptidase 1	Disruption of extracellular matrix	•		•
	TIMP-1	TIMP metallopeptidase inhibitor 1	Inhibitor of the matrix metalloproteinases	•		
	TIMP-2	TIMP metallopeptidase inhibitor 2	Inhibitor of the matrix metalloproteinases	•		
	TIMP-4	TIMP metallopeptidase inhibitor 4	Inhibitor of the matrix metalloproteinases	•		
Enzyme	ANG	Angiogenin, ribonuclease, RNase A family, 5	Angiogenesis	•		

Subsequent work by Botto et al. (2011) analyzed the secretome of infected HUVECs and identified by means of a cytokine/growth factors antibody array, 29 soluble factors, including IL-6, GM-CSF, and IL-8/CXCL8. A comparison of the HCMV secretomes derived from NHDF and HUVEC, highlighted that the presence in both secretomes of abundant cytokines/chemokines involved in AG, such as IL-6, GM-CSF, IL-8/CXCL8, CCL4/MIP-1β, CCL7/MCP-3, CCL20/MIP-3α, and CXCL11/I-TAC (Dumortier et al., 2008; Botto et al., 2011; Caposio et al., 2011, 2013). Among the EC-derived factors, IL-6 was identified as the major bioactive molecule involved in neovessel formation since its selective depletion severely decreased tubule formation (Botto et al., 2011). IL-6 is an inflammatory cytokine involved in different pathophysiological contexts, such as inflammation, lymphocytes differentiation, cell proliferation, and the inhibition of apoptotic signals (Scheller et al., 2011). IL-6 binding to its receptor (gp130) activates three different intracellular transduction pathways: (i) the JAK/STAT cascade, involved in growth regulation, survival, differentiation, and long-term inflammation-promoting effects (Horvath, 2004); (ii) the ERK1/2-MAPK pathway, and (iii) the phosphoinositol 3-kinase (PI3K)/Akt pathway, both of which are implicated in the regulation of cell growth and differentiation (Wegiel et al., 2008; Rose-John, 2012). In particular, IL-6 binding to its receptor leads to the phosphorylation of STAT3, which subsequently prevents apoptosis by blocking the activation of caspase 3 and 7, and most importantly, by increasing the expression of an apoptosis inhibitor, such as survivin (Hodge et al., 2005). Addition of the secretome derived from HCMV-infected ECs to uninfected ECs was found to stimulate expression of survivin, while antibody neutralization of IL-6 in the same secretome, abolished its capability to induce survivin or activate caspase 3 or 7 (Botto et al., 2011). Therefore, it was concluded that EC-derived HCMV secretome promotes EC survival through the expression of survivin via activation of the IL-6 pathway (Botto et al., 2011).

At the same time, in a study aimed at investigating the susceptibility of lymphatic endothelial cells (LECs) to HCMV infection, we characterized in detail the secretome of this particular type of EC (Fiorentini et al., 2011). Although, early reports have shown the presence of HCMV antigens and DNA in lymphoid tissues, the ability of HCMV to infect LECs remained unaddressed due to the lack of a suitable *in vitro* system. In this study, we observed that a clinical isolate of HCMV productively infected purified lymph node-derived LECs and dysregulated the expression of several LEC genes involved in the inflammatory response to viral infection (Fiorentini et al., 2011). Qualitative and quantitative cytokine antibody array analysis of virus-free supernatants from HCMV-infected LECs revealed the virus-induced secretion of several cytokines, chemokines, and growth factors that may be involved in the regulation of EC physiological properties. Among the released bioactive cellular proteins, the 20 most abundant included several cytokines (IL-1α, IL-3, IL-5, IL-6, IL-13, IL-15, GM-CSF, and TNF-β), several chemokines (CCL1/I-309, CCL7/MCP-3, CCL20/MIP-3α, CXCL1/GROα, CXCL5/ENA-78, CXCL9/MIG, and CXCL11/I-TAC), receptors (ICAM-1 and TNF-R1), and growth factors (TGF-β1 and bFGF). Functional assays then allowed us to establish that the secretome produced by HCMV-infected LECs indeed stimulated AG in both LECs and blood vessel ECs. Furthermore, neutralization of either IL-6 or GM-CSF in the secretome brought about the loss of its angiogenic properties. Involvement of IL-6 and GM-CSF in the HCMV-mediated lymphoangiogenesis was further supported by the finding that recombinant IL-6 and GM-CSF reproduce the same angiogenic effects as seen for the whole LEC-derived secretome. Thus, these results suggest that HCMV can stimulate both hemangiogenesis and lymphangiogenesis through an indirect mechanism that relies on the stimulation of IL-6 and GM-CSF secretion from virus-infected cells (Fiorentini et al., 2011).

A comparison of the HCMV-secretomes from different types of ECs (LECs and HUVEC) and fibroblasts (NHDF) highlights the presence of several common factors involved in both AG and WH processes, such as IL-6, GM-CSF, CCL7/MCP-3, CCL20/MIP-3α, CCL4/MIP-1β, and CXCL11/I-TAC (**Table 1**; Caposio et al., 2013).

Taking into consideration the results of the aforementioned studies, the overall effect of HCMV-derived secretomes on AG and WH in ECs is very likely related to the combination of experimental conditions and different bioactive factors, which may cooperate in a synergetic manner. In this regard, Gustafsson et al. (2015) recently observed the influence of different experimental settings, such as the cell type from which the HCMV-secretome is derived, the culture conditions, the time points of supernatant collection, and the concentration of relevant bioactive molecules, in promoting AG and WH in cultured ECs.

**FIGURE 3:**
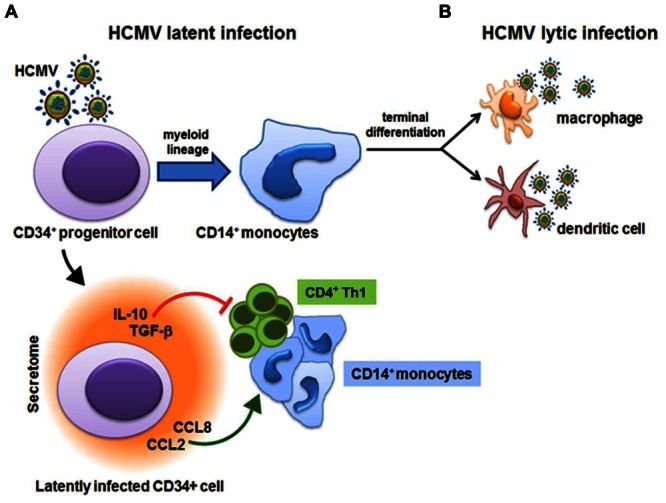
**Human cytomegalovirus (HCMV)-derived secretome and virus latency.** HCMV latency is established in bone marrow cells and is carried out in CD34^+^ hematopoietic progenitor cells and in CD14^+^ monocytes. **(A)** Latently infected CD34^+^ cells secrete CCL2/MCP-1 and CCL8/MCP-2 cytokines that recruit CD14^+^ monocytes and CD4^+^ Th1 cells to latently infected cells. However, the secretion of highly immunosuppressive cellular IL-10 and TGF-β inhibit proliferation and the antiviral functions of CD4^+^ Th1 cells. **(B)** Upon differentiation of CD14^+^ monocytes into mature macrophages or dendritic cells, reactivation of HCMV IE gene expression is promoted and the viral lytic gene program is initiated.

Whilst a number of studies have provided evidence for functional roles of HCMV-derived secretomes produced by lytically infected fibroblasts and ECs (Dumortier et al., 2008; Botto et al., 2011; Fiorentini et al., 2011; MacManiman et al., 2014), little is known about the activities of secretomes released from latently HCMV-infected cells. However, exploitation of a new experimental *in vitro* model of HCMV latency allowed Mason et al. (2012) to obtain some first insights into how latent infection is able to stimulate the release of bioactive molecules. This experimental model involves the infection of CD34^+^ hematopoietic progenitor cells cultivated in the absence of stimuli that may promote their differentiation into mature DCs or macrophages, that are fully permissive to lytic HCMV infection. These experimentally latently infected CD34^+^ cells showed long-term carriage of viral genomes in which the MIEP remained associated with transcriptionally repressive chromatin, thus hampering further HCMV lytic gene expression. These observations correlate with those made *ex vivo* of natural HCMV latent infections (Reeves et al., 2005), and provide evidence in support of the use of experimentally latently infected CD34^+^ cells as an effective model for the study of HCMV latent infection and reactivation (Reeves et al., 2005; Reeves and Sinclair, 2010). Indeed, analysis of supernatants from HCMV-infected CD34^+^ cells by large-scale cytokine antibody array has revealed the presence of many bioactive cellular proteins known to be involved in both regulation of the immune response and chemoattraction of T cells (**Table 2**). The most highly abundant cellular factors identified in this latent HCMV-secretome included: several cytokines/chemokines (XCL1/LYMPHOTACTIN, CXCL9/MIG, CXCL12/SDF-1, CXCL13/BLC, CCL1/I-309, CCL2/MCP-1, CCL8/MCP-2, CCL13/MCP-4, CCL15/MIP-1δ, CCL17/TARC, CCL20/MIP-3α, CCL27/CTACK, IL-8, IL-10/CSIF, IL-13, IL-15, LEPTIN, LTA/TNF-β, and TNF-α), growth factors (BMP-4, BMP-6, CSF-1/M-CSF, IGF-1/MGF, IGFBP3/IBP3; NT-3/NTF3, TGF-β1, and TGF-β3), a receptor (ICAM-1), and the angiogenic RNase angiogenin. However, an increase in secreted CCL2 was also observed from latently infected GMPs, and related to the stimulation of CD14^+^ monocytes migration toward the site of latency (Stern and Slobedman, 2008); whereas secretion of both CCL2 and CCL8 was measured during a short-term experimental latent infection of CD14^+^ monocytes, and linked to the recruitment of CD14^+^ monocytes and CD4^+^ Th1 cells to latently infected cells (Noriega et al., 2014). Functional analysis of the HCMV-secretome derived from latently infected CD34^+^ cells, however, highlighted that chemoattraction of CD4^+^ T cells exclusively depended on the binding of CCL8 to CC chemokine receptors CCR3 and CCR5 expressed on CD4^+^ T cells. In fact, the depletion of CD4^+^ T cells bearing CCR3 or CCR5 receptors, as well as treatment of the secretome with a neutralizing anti-CCL8 antibody, resulted in a substantially blockade of CD4^+^ T-cell migration toward latently infected cells (Mason et al., 2012). Moreover, in the same HCMV-secretome, high levels of TGF-β and cIL-10, both involved in the induction of immune tolerance by suppressing proliferation and T-cells functions were detected (Bettini and Vignali, 2009). This observation, led to the hypothesis that the secretome of latently infected CD34^+^ cells may create an immunosuppressive microenvironment, thus interfering with the immune recognition of latently infected CD34^+^ and promoting viral persistence. This hypothesis was indeed supported by the finding that TGF-β and cIL-10 in the latent secretome inhibited both the CD4^+^ Th1-mediated cytotoxicity and the production of antiviral Th1-type cytokines, such as IFN-γ, TNF-α, and TNF-β. Furthermore, treatment of latent secretome with neutralizing antibodies specific for cIL-10 and TGF-β significantly restored IFN-γ production and the cytotoxic functions of CD4^+^ cells, thus confirming that the observed inhibition of CD4^+^ T cells functions strictly depended on the secretion of TGF-β and cIL-10 by latently infected CD34^+^ cells (Mason et al., 2012). In addition, cIL-10 may prevent cell death and promote survival of latently infected cells (Weber-Nordt et al., 1996; Poole et al., 2011), since it was observed that cIL-10 is required for upregulation of the anti-apoptotic protein PEA-15 – which abolishes FAS-induced apoptosis (Poole et al., 2015). As described in Section “HCMV-Induced Secreted Cellular Proteins” above, the viral homolog LAvIL10 also stimulates the release of cIL-10 in latently infected CD34^+^ cells. Subsequently, cIL-10 may downregulate the expression of cellular microRNA hsa-miR-92a, thus inducing the secretion of the cellular CCL8 (Poole et al., 2014). Together these findings suggest that TGF-β and cIL-10 in the latent HCMV secretome by acting in a paracrine manner on uninfected bystander CD34^+^ cells, create and further expand a microenvironment conducive to the latent carriage of HCMV genomes. In fact, it was observed that uninfected CD34^+^ cells synthetized TGF-β and cIL-10, when cultured in the presence of the latent secretome (Mason et al., 2012). Overall, these findings support the existence of a complex virus strategy during latency aimed at the generation of a heavily immunosuppressive microenvironment around latently infected CD34^+^ that allows them to evade host immune recognition (**Figure 3**). In fact, even if HCMV infections leads to the recruitment of CD4^+^ T cells to site of latency through the release of CCL8, it then inhibits their antiviral functions by generating the strong immunosuppressive microenvironment, thereby avoiding the elimination of latently infected cells (Sinclair and Reeves, 2013). Furthermore, the upregulation of cIL-10 protects latently infected CD34^+^ from apoptosis, thus promoting their survival in order to maintain latent viral genome carriage (Poole et al., 2015).

**Table 2:** Main bioactive proteins assessed in the HCMV secretome of latently infected CD34+ cells.

Classification	Symbol	Gene annotation	Biological function
Cytochine/chemochine	XCL1; LTN	Chemokine (C motif) ligand 1	Leukocyte trafficking
	CXCL9; MIG	Chemokine (C-X-C motif) ligand 9	Leukocyte trafficking
	CXCL12; SDF1	Chemokine (C-X-C motif) ligand 12	Leukocyte trafficking
	CXCL13; BLC	Chemokine (C-X-C motif) ligand 13	B lymphocyte chemoattractant
	CCL1; I-309	Chemokine (C-C motif) ligand 1	Leukocyte trafficking
	CCL2; MCP-1	Chemokine (C-C motif) ligand 2	Leukocyte trafficking
	CCL8; MCP-2	Chemokine (C-C motif) ligand 8	Leukocyte trafficking
	CCL13; MCP-4	Chemokine (C-C motif) ligand 13	Leukocyte trafficking
	CCL15; MIP-1δ	Chemokine (C-C motif) ligand 15	T-cells and monocyte chemoattractant
	CCL17; TARC	Chemokine (C-C motif) ligand 17	T cells chemotaxis and trafficking
	CCL20; MIP-3α	Chemokine (C-C motif) ligand 20	Leukocyte trafficking
	CCL27; CTACK	Chemokine (C-C motif) ligand 27	T-cell-mediated inflammation, T lymphocyte chemoattractant
	IL-8	Interleukin-8	Leukocyte trafficking
	IL-10; CSIF	Interleukin-10	Anti-inflammatory cytokine
	IL-13	Interleukin-13	Anty-inflammatory cytokine
	IL-15	Interleukin-15	T and natural killer cells activation and proliferation
	LEP	Leptin	Angiogenesis and wound healing
	LTA; TNF-β	Lymphotoxin A	Proinflammatory cytokine
	TNF-α	Tumor necrosis factor alpha	Proinflammatory cytokine
Growth factor	BMP-4	Bone morphogenetic protein 4	Bone and cartilagine development
	BMP-6	Bone morphogenetic protein 6	Bone and cartilagine growth
	CSF-1; M-CSF	Colony stimulating factor 1	Monocytes proliferation, differentiation, and survival
	IGF-1; MGF	Insulin-like growth factor 1	Cell growth and development
	IGFBP3; IBP3	Insulin-like growth factor binding protein 3	Cell growth and development
	NT-3; NTF3	Neurotrophin-3	Neurogenesis
	TGF-β1	Transforming growth factor beta 1	Cell growth, proliferation, differentiation, adhesion and migration
	TGF-β3	Transforming growth factor beta 3	Cell adhesion, proliferation, differentiation
Receptor	ICAM-1	Intercellular adhesion molecule 1	Cell adhesion for endothelial and immune system cells
Enzyme	ANG	Angiogenin, ribonuclease, RNase A family	Angiogenesis

Although, HCMV lytic and latent host-cell interactions involve different target cell types and viral genetic programs, and lead to different outcomes, many bioactive factors are common between the HCMV-induced secretome derived from lytically and latently infected cells. In particular, various cytokines/chemokines, such as CXCL9/MIG, CCL1/I-309, CCL8/MCP-2, CCL15/MIP-1δ, CCL20/MIP-3α, IL-8, IL-10, IL-13, IL-15, LTA/TNF-β, and TNF-α, the growth factor TGF-β, the receptor ICAM-1, and the enzyme ANG are present in both lytically- and latently-infected cells secretomes. However, the presence of a set of common bioactive factors between the two functionally different secretomes is of great interest considering the opposite outcomes of both forms of infections; indeed, we might hypothesize that some of the pathophysiological alterations of the microenvironment surrounding HCMV-infected cells that are induced by the bioactive molecules that the two secretomes have in common, may occur during both lytic and latent infection.

### Secreted HCMV Proteins

Many viral-encoded secreted bioactive proteins have been identified in the supernatants of herpesvirus-infected cells. Among them, cytokine-like proteins, such as the viral counterparts of cellular IL-10, IL-17, and IL-6 were found in the supernatants from cells infected with Epstein–Barr virus (EBV; Ryon et al., 1993; Swaminathan et al., 1993), herpesvirus saimiri (Yao et al., 1995), or human herpesvirus-8 (HHV-8; Neipel et al., 1997), respectively. In this section, we review the main biochemical and functional features of the HCMV-encoded proteins that have identified to date within the secretome of infected cells.

#### UL21.5

This was the first protein of viral origin identified as a secreted product in the supernatant of HCMV-infected cells (Müllberg et al., 1999). UL21.5 mRNA is abundantly expressed at the late stage of HCMV infection (Rawlinson and Barrell, 1993) and is transcribed by two exons; the first encodes a signal peptide of 20 amino acids followed by an additional sequence of eight amino acids, while the second exon codes the remaining 75 residues. Intriguingly, UL21.5 mRNA is also packaged into virions, thus suggesting a role of the encoded protein even before the infecting viral genome reaches the nucleus and becomes transcriptionally active (Bresnahan and Shenk, 2000).

Later, it was demonstrated that pUL21.5 is a soluble CC chemokine receptor able to selectively bind CCL5/RANTES with high affinity and thus preventing its interaction with cellular receptors (Wang et al., 2004). pUL21.5 by acting as a secreted receptor decoy even at a distance from infected cells, may therefore counteract chemokine-mediated host antiviral response (Wang et al., 2004).

#### UL146 and UL147

UL146 and UL147 proteins are considered as potential viral encoded CXC-chemokines due to the presence of putative signal sequences, cysteine spacing, and (in pUL146 only) an ELR-CXC sequence (glutamic acid -E-, leucine -L-, arginine -R-, nominated the ELR sequence, upstream of the CXC motif). Such features are common to cellular CXC cytokines, such as IL-8 and Gro-α (Penfold et al., 1999; McSharry et al., 2012). Sequence analysis of UL146 and UL147 proteins from the Toledo strain of HCMV reveals 24 and 16% amino acid identity to IL-8 (McSharry et al., 2012), respectively; accordingly, UL146 protein is also known as vCXCL-1, and UL147 protein as vCXCL-2.

While the functions of UL147 have not yet been characterized, light has been shed on those of UL146 (Miller-Kittrell et al., 2007; Heo et al., 2015). vCXCL-1 (UL146) is a 117 amino acids glycoprotein secreted into the culture medium with late kinetics. The UL146 gene product is highly polymorphic among low-passage strains (Heo et al., 2008) and absent in laboratory strains of the virus, such as AD169 and Towne (Cha et al., 1996). Recently, it was observed that vCXCL-1 is able to: (i) induce calcium flux via CXCR2 (Heo et al., 2015); (ii) selectively stimulate the expression of beta2 integrin (CD11b and CD11c), responsible for the adherence of leukocytes to the vascular endothelium through an interaction with ICAM-1 and for modulating the life span of neutrophils (Mayadas and Cullere, 2005; Heo et al., 2015); (iii) induce the migration of neutrophils *in vitro* (Heo et al., 2015); and (iv) upregulate CCL22, a chemokine involved in chemotaxis in monocytes, dendritic and NK cells, and chronically activated T lymphocytes (Heo et al., 2015).

Together, the capability of vCXCL-1 to alter the trafficking of HCMV-infected neutrophils, as potential HCMV carriers, may facilitate viral dissemination and promote the spread of HCMV through the host (McSharry et al., 2012).

#### UL128

The UL128 protein is part of the pentameric complex composed of gH (pUL115), gL (pUL75), pUL130, and pUL131A that is required for HCMV entry into epithelial, endothelial and DCs (Revello and Gerna, 2010). However, pUL128 contains four conserved cysteine amino acids at its N-terminus, similar to those found in the CC-chemokines (Akter et al., 2003). To this regard, it was observed that UL128 is able to induce the migration of human PBMCs (peripheral blood mononuclear cells) to levels comparable to those induced by the human CCL3/MIP-1α chemokine (Zheng et al., 2012). In addition, treatment with soluble pUL128 increases the expression of both IL-6 and TNF-α in PBMCs, and stimulates PBMCs proliferation through activation of the MAPK pathway (Zheng et al., 2012). Alteration in PBMCs trafficking and cytokines secretion by soluble pUL128 may thus facilitate HCMV dissemination through the recruitment of infected carrier cells.

#### IL-10

Among the HCMV-encoded cytokine-like molecules, the latency-associated viral cellular IL-10 homologu (LAcmvIL-10), encoded by HCMV UL111A gene is worthy of note (Ouyang et al., 2014). This HCMV gene encodes two IL-10 homologs generated by alternative splicing (Kotenko et al., 2000; Jenkins et al., 2004): cmvIL-10 of 175 amino acids, expressed during productive infection with late kinetics (Spencer et al., 2002; Chang et al., 2004), and LAcmvIL-10, of 139 amino acids, the result of a C-terminal truncation, reported to be expressed with early kinetics during both productive and latent infection (Jenkins et al., 2004).

As the cellular IL-10 (cIL10), cmvIL-10, and LAcmvIL-10 downregulate the expression of MHC-II molecules in latently infected GMPs (Spencer et al., 2008). However, cmvIL-10 shares more functions in common with cIL-10 than LAcmvIL-10 (Jenkins et al., 2008b). In fact, cmvIL-10, but not LAcmvIL-10, increases the expression of the IgG (FCγ) receptors CD32 and CD64, and increases FCγ receptor-mediated phagocytosis (Jaworowski et al., 2009). Similar to cIL-10, cmvIL-10 then inhibits the expression of proinflammatory cytokines in LPS-stimulated MDDCs (Monocyte-Derived Dendritic Cells; Jenkins et al., 2008a,b). Concerning the role of LAcmvIL-10, it was recently observed that its expression in latently infected CD34^+^ cells resulted in the downregulation of cellular microRNA hsa-miR-92a, which upregulates the myeloid transcription factor GATA2 (Poole et al., 2013, 2014). GATA2, in turn, increases the transcription of the latency-associated HCMV genes LUNA and UL144 (Reeves, 2011; Poole et al., 2013), as well as the transcription of the cellular IL-10 (Poole et al., 2011). Since cIL-10 is one of the most abundant cytokines in the secretome produced by cells latently infected with HCMV, and believed to create an immune suppressive environment and to suppress apoptosis (Mason et al., 2012; Poole and Sinclair, 2015), LAcmvIL-10 is thought to reduce the ability of CD4^+^ cells to recognize HCMV-infected cells during latent infection (Mason et al., 2012; Poole and Sinclair, 2015).

#### UL7

More recently, a novel HCMV-encoded secreted molecule, the protein encoded by UL7 gene, has been identified as a critical component of the HCMV-secretome responsible for vascular dysregulation associated with persistent HCMV infection (MacManiman et al., 2014). The UL7 gene belongs to the RL11 family, located in the left end of the viral genome, and characterized by early-late-phase kinetics during lytic HCMV infection (Engel et al., 2011). Structure prediction of UL7 protein identified a 222-amino-acid type I glycoprotein characterized by a putative leader peptide (35 amino acids), an extracellular immunoglobulin superfamily domain (Ig-like domain; 102 amino acids), a mucine-like stalk region (55 amino acids), a hydrophobic transmembrane sequence (22 amino acids) and a short cytoplasmic tail (eight amino acids; Engel et al., 2011). Engel et al. (2011) observed that the UL7 protein is proteolytically cleaved in correspondence with the stalk region, resulting in a heavily glycosylated ectodomain that is released from infected cells. Interestingly, the UL7 Ig-like domain shares significant amino acid identity with both CD229 (member of SLAM family), which is involved in T-cell signaling (Engel et al., 2011), and the carcinoembryonic antigen-related cell adhesion molecule (CEACAM) protein, that is highly expressed during vasculogenesis (MacManiman et al., 2014). However, UL7 is not able to bind directly CD229 or any other member of the SLAM family, but it can contact a putative UL7 ligand on the surface of monocyte-derived DCs, thus interfering with pro-inflammatory responses (Engel et al., 2011). In particular, UL7 has been observed to downregulate the production of pro-inflammatory cytokines, such as TNF, IL-6, and IL-8 in primary human monocyte-derived DCs and in the PMA-induced myeloid cell lines, U937 and THP-1 (Engel et al., 2011). These findings have thus suggested UL7 as a novel HCMV-encoded product able to contribute to the inhibition of the host antiviral defense in favor of the establishment of persistent infection (Engel et al., 2011). Moreover, the activity of CEACAM-1 in promoting vasculogenesis led MacManiman et al. (2014) to hypothesize a potential role of UL7 in this process. In support of this, using *in vitro* AG assays, they found that the secretome generated by an UL7-deficient HCMV produced a 50% reduction in tubulogenesis compared with that for the secretome from wild-type virus. Furthermore, following adenovirus-mediated transduction of human aortic endothelial cells (HEACs) with either the full length UL7 or the UL7 ectodomain, they observed the appearance of a robust network of interconnecting tubules, thus sustaining the involvement of UL7, and in particular its ectodomain, in the promotion of ECs differentiation (MacManiman et al., 2014). Moreover, the observation of an increased secretion of IL-6 and the activation of STATs and MAPK pathways in ECs overexpressing UL7 further substantiates the role of UL7 in angiogenesis (MacManiman et al., 2014). Taken together, these findings support a role of secreted UL7 protein in regulating some physiological properties of ECs and in the stimulation of angiogenesis.

### The Importance of HCMV-Induced Bioactive Molecules in Virus Pathogenesis

Human cytomegalovirus infections are associated with the acceleration of various long-term vascular diseases, especially in immunocompromised hosts (Griffiths et al., 2015). In these settings, HCMV has been correlated to the acceleration of a number of vascular diseases, such as atherosclerosis, restenosis, and TVS, which are all determinants of chronic rejection (CR), one of the most important long-term conditions leading to graft failure and re-transplantation (Grahame-Clarke, 2005; Streblow et al., 2007). TVS is a complex phenomenon in which the leading event is a diffuse and concentric intimal proliferation, which, in turn, determines vessel occlusion due to perivascular inflammation, ECs dysfunction, and hvSMC proliferation with extracellular matrix deposition. In addition, the progression of TVS is promoted by the same mechanisms that lead to AG and WH (Streblow et al., 2007, 2008). In early TVS lesions, macrophages, T cells, B cells, and NK cells are present, while progression to late lesions is associated with a thinning intima containing SMC and macrophages (Streblow et al., 2005). Strong correlations between HCMV reactivation and the acceleration of vascular diseases development have been made on the basis of: (i) the high efficiency rate of infection for those cell types involved in TVS, including EC, SMC, and monocyte-derived macrophages (Lemström et al., 1993); (ii) the delay in the TSV progression and a prolonged graft survival in transplant recipients after treatment with ganciclovir, an approved anti-HCMV drug (Lemström et al., 1997); and (iii) *in vivo* studies on CMV animal models (Lemström et al., 1997; Streblow et al., 2003). In particular, in a rat model of aortic allografts, it was observed that infection with rat cytomegalovirus (RCMV) accelerates atherosclerosis, leading to an overall rearrangement of both the structural and functional architecture of vessel cells (Lemström et al., 1993; Streblow et al., 2003), the reversal of these effects through the use of an anti-CMV agent, such as ganciclovir, further support a role of the virus in the pathogenesis of experimental atherosclerosis (Lemström et al., 1994, 1997). RCMV infection has also been associated with an altered profile of released cytokines, in which higher levels of IL-2 and IL-4 were evident compared with controls (Zhou et al., 1999).

Furthermore, the observation that the HCMV-secretomes from both fibroblasts and ECs stimulate AG and WH in *in vitro* models suggests that HCMV infection of the allograft in transplant recipients and the subsequent release of a virus-altered secretome may promote AG and WH in the host, thus resulting in the acceleration of TVS (Streblow et al., 2008; Caposio et al., 2011, 2013). On the other hand, the stimulation of “*de novo*” lymphatic and blood vessels sprouting may represent a strategy adopted by the virus to enhance its replication and dissemination from the initial infection site to other host tissues.

In contrast, the available data about the activities of the HCMV-derived secretome from latently infected cells, indicate that the generation of a secretome-induced immunosuppressive microenvironment around latently infected cells may help the virus escape recognition by the hosts’s immune system, thus favoring cell survival, viral persistence and potential reactivations.

Clearly, in both lytic and latent infections, the activities of the wide range of bioactive molecules in HCMV-secretomes may have severe pathogenetic consequences since they facilitate both virus dissemination and persistence in the host in spite of a robust antiviral response.

## Conclusion

One of the main intriguing features of HCMV biology is the exceptionally wide arsenal of virus-encoded proteins that have been observed to be capable of counteracting host innate and adaptive immune defenses. Persistence of the virus, even in the immunocompetent host, and its ability to avoid immune clearance may be due in part to the activities of the many viral immunomodulatory proteins (Mocarski et al., 2014). In this review, we have examined what is known about the bioactive molecules, of both viral and cellular origin, secreted from HCMV-infected cells. These proteins are able to modify the extracellular environment of host infected tissues, thus promoting both virus dissemination and persistence, as well as some pathophysiological processes, such as AG and WH, that may drive the development of associated vascular diseases. However, more work is needed to fully appreciate the importance of HCMV-derived secretomes in the pathogenesis of HCMV diseases.

To date, ECs, fibroblasts, and hematopoietic precursor cells have been mainly used to investigate the composition and functions of HCMV-derived secretomes; but, in the future, a deeper understanding of the importance of the HCMV secretome may come from the analysis of secretomes derived from other cell types. For example, little is known about the impact of HCMV infection on the secretion of bioactive molecules from epithelial cells, which represent one of the main cell targets for productive infection in the natural host. Does the HCMV-induced secretome of epithelial cells able to alter the host’s immune recognition of infected cells or to catalyze organ damage in relation to lytic infection as occurs in the retina (Momma et al., 2003)?

The findings that we have reviewed here have led to a better comprehension of both the viral and cellular bioactive molecules involved in the host-HCMV relationship. Little is known, however, about the molecular mechanisms underlying the modulation and secretion of such cellular bioactive proteins. For example, the HCMV mechanisms that interfere directly or indirectly with the cellular secretion pathways remain to be identified. Knowledge of these molecular dynamics would be useful for the development of strategies aimed at attenuating or blocking the ability of HCMV to interfere with the host immune responses that contributes to the pathological outcome. Finally, it will also be necessary to confirm the *in vitro* findings in *in vivo* models, such as a humanized mouse model. The confirmation of experimental data and hypotheses would support the rationale to deepen our understanding of HCMV immunomodulation in order to identify targets for novel therapeutic strategies to prevent or control HCMV diseases.

## Author Contributions

AL wrote the manuscript; MT wrote the manuscript; and GG conceived of the review and wrote the manuscript.

## Conflict of Interest Statement

The authors declare that the research was conducted in the absence of any commercial or financial relationships that could be construed as a potential conflict of interest.
